# 基于三甲基苯磺酰羟胺消除反应的氧连接氮乙酰葡萄糖胺修饰肽段的精准鉴定

**DOI:** 10.3724/SP.J.1123.2020.12024

**Published:** 2021-11-08

**Authors:** Zhixin GUO, Hang LI, Weijie QIN

**Affiliations:** 军事科学院军事医学研究院生命组学研究所, 北京蛋白质组研究中心, 国家蛋白质科学中心(北京), 蛋白质组学国家重点实验室, 北京 102206; Institute of Lifeomics, Academy of Military Medical Sciences, Academy of Military Sciences,Beijing Proteome Research Center, National Center for Protein Sciences (Beijing),State Key Laboratory of Proteomics, Beijing 102206, China; 军事科学院军事医学研究院生命组学研究所, 北京蛋白质组研究中心, 国家蛋白质科学中心(北京), 蛋白质组学国家重点实验室, 北京 102206; Institute of Lifeomics, Academy of Military Medical Sciences, Academy of Military Sciences,Beijing Proteome Research Center, National Center for Protein Sciences (Beijing),State Key Laboratory of Proteomics, Beijing 102206, China; 军事科学院军事医学研究院生命组学研究所, 北京蛋白质组研究中心, 国家蛋白质科学中心(北京), 蛋白质组学国家重点实验室, 北京 102206; Institute of Lifeomics, Academy of Military Medical Sciences, Academy of Military Sciences,Beijing Proteome Research Center, National Center for Protein Sciences (Beijing),State Key Laboratory of Proteomics, Beijing 102206, China

**Keywords:** 富集鉴定, 代谢标记, 氧连接氮乙酰葡萄糖胺, 半胱氨酸巯基-叠氮糖人为修饰物, 三甲基苯磺酰羟胺, enrichment and identification, metabolic labeling, *O*-linked *β*-*N*-acetylglucosamine (*O*-GlcNAc), cysteine thiol-azidosugar artificial modification, *O*-mesitylenesulfonylhydroxylamine (MSH)

## Abstract

氧连接氮乙酰葡萄糖胺(*O*-GlcNAc)是一种重要的蛋白质翻译后修饰,它在维持机体正常的生命活动中发挥着重要作用。许多研究证实,*O*-GlcNAc糖基化修饰稳态的破坏与人类多种疾病的发生相关,大规模富集鉴定*O*-GlcNAc糖基化修饰蛋白有助于发现新的临床疾病诊断标志物。由于*O*-GlcNAc糖基化修饰丰度较低,形成的糖苷键不稳定,*O*-GlcNAc糖基化修饰蛋白/肽段的富集鉴定面临一定挑战。近年来,全乙酰化的非天然糖代谢标记技术被广泛应用于*O*-GlcNAc糖基化修饰蛋白/肽段的富集鉴定。然而,最新的研究发现,在细胞代谢标记过程中,全乙酰化的非天然单糖会同时标记半胱氨酸的巯基而引入半胱氨酸巯基-叠氮糖人为修饰物。该副反应在一定程度上干扰了*O*-GlcNAc糖基化修饰蛋白/肽段的富集鉴定。鉴于此,研究发展了一种通过三甲基苯磺酰羟胺(MSH)特异性氧化消除半胱氨酸巯基-叠氮糖人为修饰物的方法,进而显著提高*O*-GlcNAc糖基化修饰肽段的精准鉴定。该方法建立于温和的磷酸钠缓冲液(50 mmol/L, pH=8)体系,利用过量的MSH,于95 ℃避光振荡反应30 min,可完全消除半胱氨酸巯基-叠氮糖人为修饰物。该方法应用于Hela细胞中,可有效消除叠氮全乙酰化半乳糖胺(Ac_4_GalNAz)代谢产生的半胱氨酸巯基-叠氮糖人为修饰物,从而成功富集鉴定到157条*O*-GlcNAc糖基化修饰肽段,归属于130个蛋白质。该方法有效去除了半胱氨酸巯基-叠氮糖人为修饰物对代谢标记结果的干扰,为非天然糖代谢标记技术在糖蛋白组学分析中的应用提供了新的研究策略。

## Abstract (en)

*O*-linked *β*-*N*-acetylglucosamine (*O*-GlcNAc), an important post-translational modification of proteins, plays an essential role in maintaining normal biological activities of organisms. Studies have shown that the disruption of *O*-GlcNAc homeostasis is closely associated with various human diseases. Therefore, large-scale enrichment and identification of *O*-GlcNAc proteins is important for exploring diagnostic biomarkers of clinical diseases. *O*-GlcNAcylation is substoichiometric, and its glycosidic bond is unstable; hence, the enrichment and identification of *O*-GlcNAc proteins remains a challenge. Recently, metabolic labeling technology with per-*O*-acetylated unnatural monosaccharides has been introduced to enrich *O*-GlcNAc proteins or peptides in cells. However, azidosugars can also react with the thiol group of cysteine to form cysteine thiol-azidosugar artificial modification as a byproduct in cell metabolism, which interferes with the identification of *O*-GlcNAc proteins or peptides. Therefore, the development of a methodology for the specific and complete removal of cysteine thiol-azidosugar artificial modification is necessary. *O*-Mesitylenesulfonylhydroxylamine (MSH) is an oxidizing and aminating reagent with great potential to oxidatively eliminate the cysteine thiol modification to dehydroalanine. Hence, it is worthwhile to probe whether MSH can eliminate cysteine thiol-azidosugar artificial modification. Two cysteine thiol-azidosugar artificial modifications were synthesized successfully by incubating thiol standard peptides with tetraacetylated *N*-azidoacetylgalactosamine (Ac_4_GalNAz) in sodium carbonate buffer (200 mmol/L, pH 10) at 37 ℃ for 90 min. Then, the reaction conditions for MSH oxidative elimination were optimized to completely remove cysteine thiol-azidosugar artificial modification. Sodium phosphate buffer (50 mmol/L, pH 8.0) was selected to protect the *O*-GlcNAc modification due to its mild nature. After extensive investigation, the optimized reaction conditions were established as 95 ℃ (reaction temperature) and 30 min (reaction time). Both cysteine thiol-azidosugar artificial modifications could be removed completely under these conditions. Furthermore, two azide-labeled *O*-GlcNAc (N_3_-*O*-GlcNAc) peptides were used to assess whether MSH destroyed the *O*-GlcNAc modification at the same time. The results showed that the two N_3_-*O*-GlcNAc peptides were stable after treatment with MSH at 95 ℃ for 30 min. In short, with excess MSH, the N_3_-*O*-GlcNAc peptides were stable, but the cysteine thiol-azidosugar artificial modification was exhausted in sodium phosphate buffer (50 mmol/L, pH 8.0) at 95 ℃ for 30 min. Moreover, both *O*-GlcNAc modification and cysteine thiol-azidosugar artificial modification exist in cell metabolism. This method can not only remove cysteine thiol-azidosugar artificial modification but also ensure the stability of *O*-GlcNAc modification. There is growing evidence that *O*-GlcNAcylation mainly occurs in proteins in the cytoplasm and nucleus, and that most *O*-GlcNAc proteins are involved in important biological signaling pathways. The nuclear and cytoplasmic proteins incubated with Ac_4_GalNAz in HeLa cells were selected as a model system. After the nuclear and cytoplasmic proteins were digested into peptides, MSH was applied to remove cysteine thiol-azidosugar modification. In addition, 51 peptides for the elimination of the cysteine thiol-azidosugar artificial modification were identified, indicating that MSH can remove the interference of cysteine thiol-azidosugar artificial modification in cell metabolism. Biotin probe and streptavidin dynabeads were subsequently used to label and enrich N_3_-*O*-GlcNAz peptides in cell metabolism. Finally, 157 *O*-GlcNAc peptides attributed to 130 proteins were identified. To better understand the functional roles of *O*-GlcNAc proteins, gene ontology analysis was performed. Cell component analysis showed that the identified *O*-GlcNAc proteins were mainly distributed inpostsynaptic density, cytoplasm, and condensed nuclear chromosome. The proteins were mostly involved in biological processes, including cell division, excitatory postsynaptic potential, and microtubule-based movement. The proteins responsible for transferase activity, transferring acyl groups, histone acetyltransferase activity, and microtubule binding were highly enriched, indicating that *O*-GlcNAc proteins play important roles in cells. In summary, this work developed an approach to enrich *O*-GlcNAc peptides precisely in metabolic labeling by removing cysteine thiol-azidosugar artificial modification with MSH. This methodology provides a new strategy for the application of metabolic labeling technology with unnatural monosaccharides in glycoproteomics analysis.

氧连接氮乙酰葡萄糖胺(*O*-GlcNAc)是一种重要的蛋白质翻译后修饰,它广泛参与机体的众多生命活动,而其稳态的破坏与人类多种疾病的发生发展密切相关,如糖尿病、神经退行性疾病、癌症等^[1,2,3,4,5,6,7]^。大规模富集鉴定*O*-GlcNAc修饰蛋白有助于深度挖掘其生物学功能及相关疾病机制。由于*O*-GlcNAc糖基化修饰丰度低、形成的糖苷键不稳定等^[8]^, *O*-GlcNAc糖基化修饰蛋白/肽段的富集鉴定面临一定挑战。因此,发展高效的*O*-GlcNAc糖基化修饰蛋白/肽段富集鉴定新方法是对其深入研究的关键。

非天然糖代谢标记技术作为蛋白质糖基化研究的通用技术被广泛应用于*O*-GlcNAc糖基化修饰蛋白/肽段的富集鉴定^[9,10,11,12]^。非天然糖通常携带一个生物正交化学报告基团(如叠氮),其亲水性羟基常以疏水性的乙酰基包裹保护,从而增强非天然糖的细胞摄取。全乙酰化的非天然糖进入细胞后通过细胞内糖代谢途径转化为相应底物尿苷二磷酸-氮乙酰葡糖胺(UDP-GlcNAz),进而被*O*-GlcNAc转移酶(OGT)识别并整合在目标蛋白质的丝氨酸或苏氨酸羟基上,随后可通过特异性的生物素探针捕捉目标蛋白质,实现*O*-GlcNAc糖基化修饰蛋白/肽段的富集鉴定^[13]^。然而,最新研究表明,在细胞代谢标记过程中,全乙酰化的非天然糖会同时标记半胱氨酸的巯基而产生半胱氨酸巯基-叠氮糖人为修饰物^[14,15,16,17]^,并且在蛋白质或肽段层面的*O*-GlcNAc糖基化修饰鉴定结果中,难以排除副反应产物半胱氨酸巯基-叠氮糖人为修饰物的干扰。因此,建立一种特异性去除半胱氨酸巯基-叠氮糖人为修饰物的方法具有十分重要的意义。

近年来,三甲基苯磺酰羟胺(MSH)被广泛应用于蛋白质半胱氨酸巯基修饰物的氧化消除。MSH是一种氧化及胺化试剂,它可以提供游离的氨基以进攻硫醚中的亲核性硫原子,在一定碱性条件下转化为亚硫亚胺中间体,进而发生消除反应生成烯烃产物^[18]^。Davis课题组^[19]^在pH=8的温和体系下,利用MSH氧化消除丝氨酸蛋白酶突变体*S*-乙基半胱氨酸中的硫醚键,成功将其转化为脱氢丙氨酸。随后,MSH氧化消除法又被成功应用于半胱氨酸巯基-微囊藻毒素修饰物中硫醚键的断裂^[20]^。在此基础上,本研究发展了MSH特异性消除半胱氨酸巯基-叠氮糖人为修饰物的方法,实现了细胞中*O*-GlcNAc糖基化修饰肽段的精准鉴定,为非天然糖代谢标记技术在糖蛋白组学分析中的应用提供了新的研究策略。

## 1 实验部分

### 1.1 仪器与试剂

Ultrafle Xtreme MALDI TOF/TOF基质辅助激光解析飞行时间质谱仪(Bruker公司,德国), EASY Nlc 1200 Orbitrap Fusion Lumos质谱仪、HERAcell VIOS 160i细胞培养箱、MSC-100 Thermo-Shaker恒温振荡金属浴、Micro 21微量离心机、Nano Drop 2000C超微量分光光度计(Thermo Fisher Scientific公司,美国), Concentrator Plus真空离心浓缩仪(Eppendorf公司,德国), Sartorius BP211d分析天平(Sartorius公司,德国), LX-200型迷你离心机(海门市其林贝尔仪器制造有限公司,江苏)。

细胞低糖培养基购自美国Invitrogen公司。*O*-GlcNAc水解酶抑制剂(Thiamet G)购自美国Santa Cruz Biotechnology公司。磷酸盐缓冲溶液(PBS)购自美国Corning公司。二甲基亚砜(DMSO)购自北京百灵威科技有限公司。叠氮全乙酰化半乳糖胺(Ac_4_GalNAz)、核质蛋白提取试剂盒、三苯基磷-生物素探针、链霉亲和素磁珠和表面活性剂去除柱均购自美国Thermo Fisher Scientific公司。十二烷基磺酸钠(SDS)购自美国USB公司。MSH购自上海易恩化学技术有限公司。盐酸三(羟甲基)氨基甲烷(Tris-HCl, 1 mol/L, pH=7.4)购自北京索莱宝科技有限公司。碳酸氢铵(NH_4_HCO_3_)、甲酸(FA)、乙腈(ACN)、三氟乙酸(TFA)、2,5-二羟基苯甲酸(DHB)和乙基苯基聚乙二醇(NP-40)均购自德国Sigma Aldrich公司。磷酸二氢钠(NaH_2_PO_4_)、磷酸氢二钠(Na_2_HPO_4_)、碳酸氢钠(NaHCO_3_)、氯化钠(NaCl)、碳酸钠(Na_2_CO_3_)和*N*,*N*-二甲基甲酰胺(DMF)均购自上海国药集团化学试剂有限公司。所有化学试剂均为分析级或HPLC级,不经额外纯化直接使用。

### 1.2 MSH氧化消除反应的建立

1.2.1 半胱氨酸巯基-叠氮糖人为修饰物的制备

首先取30 μg巯基标准肽段溶于23 μL碳酸钠缓冲液(25 mmol/L, pH=10)中,然后加入终浓度2 mmol/L的Ac_4_GalNAz,于37 ℃恒温箱孵育90 min,然后经C18柱脱盐热干备用,待MALDI-TOF MS分析。

1.2.2 MSH氧化消除

将上述制备的半胱氨酸巯基人为修饰物复溶于15 μL磷酸钠缓冲液(50 mmol/L, pH=8),测定浓度后取出4 μg加入磷酸钠缓冲液(50 mmol/L, pH=8)至终体积40 μL,取出5 μL作为实验对照组备用。

将1 μg叠氮标记的*O*-GlcNAc(N_3_-*O*-GlcNAc)标准肽段溶于磷酸钠缓冲液(50 mmol/L, pH=8)中,配至终体积40 μL,取出5 μL作为实验对照组备用。

剩余样品中分别加入终浓度15 mmol/L的MSH,于室温快速涡旋1 min后,随后置于95 ℃避光振荡反应30 min,反应前后均采用MALDI-TOF MS进行分析。

### 1.3 Hela细胞中*O*-GlcNAc修饰肽段的富集

1.3.1 Hela细胞代谢培养

待Hela细胞生长状态良好时进行代谢培养:在10 mL细胞低糖培养基中加入终浓度100 μmol/L的Ac_4_GalNAz,再加入终浓度10 μmol/L的Thiamet G备用。移去Hela细胞的原培养基,用PBS清洗细胞两次,加入配制的低糖培养基于37 ℃培养箱培养24 h。

1.3.2 核质蛋白提取及酶切

收集的细胞于4 ℃以2000 g离心3 min后移去上清液,使用核质蛋白提取试剂盒提取核质蛋白。首先向细胞加入1000 μL细胞质蛋白提取试剂I,以2500 r/min涡旋15 s悬浮沉淀,于冰上放置10 min后加入55 μL细胞质蛋白提取试剂II,以2500 r/min涡旋5 s,再于冰上放置1 min,以2500 r/min涡旋5 s,随后于4 ℃以16000 g离心5 min,转移上清胞质提取物至离心管。在剩下的组分中加入500 μL细胞核蛋白提取试剂,以2500 r/min涡旋15 s,冰上放置10 min,重复操作4次,于4 ℃以16000 g离心10 min,转移上清至离心管,合并两次上清液,即为核质蛋白。

将提取的核质蛋白转移至10 kDa超滤管,以14000 g离心15 min,用50 mmol/L NH_4_HCO_3_溶液清洗4次,检测蛋白质浓度。在核质蛋白中加入胰蛋白酶(胰蛋白酶与蛋白质的质量比为1:50),于37 ℃恒温箱酶切16 h,酶切后以14000 g离心10 min,收集肽段,并用水清洗超滤管两次。收集的肽段于45 ℃真空热干后置于-80 ℃冰箱储存备用。

1.3.3 酶切肽段的氧化消除

热干的肽段用磷酸钠缓冲液(50 mmol/L, pH=8)复溶,并测定其浓度。随后将肽段溶液稀释至1 μg/μL,取400 μL加入终浓度15 mmol/L的MSH,室温快速涡旋1 min后于95 ℃避光振荡反应30 min。反应完成后用C18柱脱盐,于45 ℃真空热干备用。

1.3.4 N_3_-*O*-GlcNAc修饰肽段的富集及洗脱

取链霉亲和素磁珠50 μL,弃去保护液,用300 μL PBS清洗4次,备用。热干的肽段用100 μL PBS复溶,加入终浓度200 μmol/L的三苯基磷-生物素探针于37 ℃恒温箱孵育过夜,标记N_3_-*O*-GlcNAc修饰肽段。使用0.5 mL表面活性剂去除柱去除未反应的三苯基磷-生物素探针。生物素标记后的肽段补加PBS至终体积672 μL,并加入28 μL含100 g/L SDS的水溶液,然后将其全部转移至磁珠中,室温旋转孵育1 h,孵育结束后,弃去上清液。

称取146.2 mg NaCl和70.9 mg Na_2_HPO_4_,溶于25 mL水中,并加入25 mL Tris-HCl,配制为缓冲液A;依次使用300 μL含0.2% (v/v)NP-40的缓冲液A、300 μL缓冲液A及300 μL水分别清洗磁珠2次;然后加入80 μL含0.15% (v/v)TFA的水溶液,于95 ℃煮10 min,取洗脱液于离心管中;继续用80 μL含0.15% (v/v) TFA的水溶液及80 μL洗脱液TFA-ACN-H_2_O(1:50:49, v/v/v)分别清洗磁珠1次;洗脱液合并过C8膜后于45 ℃真空热干,热干后置于-80 ℃冰箱储存备用。

### 1.4 分析条件

1.4.1 MALDI-TOF MS

将DHB溶于H_3_PO_4_-ACN-H_2_O(1:29:70, v/v/v)溶液,配制成25 mg/mL的DHB基质溶液。将1 μL样品及1 μL DHB基质溶液混合点于靶板上后室温干燥,采用阳离子反射模式在20 kV加速电压下进行谱图采集。

1.4.2 LC-MS/MS

流动相A和B分别为含0.1%(v/v) FA的水溶液和FA-H_2_O-ACN (1:19:80, v/v/v)。富集的肽段用流动相A复溶,以600 nL/min通过C18反相分析柱(150 mm×0.15 mm, 1.9 μm)实现样品分离。洗脱程序为:0~8 min, 6%B~12%B; 8~58 min, 12%B~30%B; 58~70 min, 30%B~40%B; 70~71 min, 40%B~95%B; 71~78 min, 95%B。

喷雾电压设定为2.3 kV,质谱分析的一级扫描范围设置为300~1400 Da,分辨率为120000;质谱的二级谱图以数据依赖型扫描模式(DDA)进行采集,二级碎裂方式为高能诱导解离(HCD),能量设置为35%,离子注入时间为50 ms。

### 1.5 数据检索

LC-MS/MS数据(Raw文件)使用MaxQuant(版本1.6.17.0)以UniProt人类蛋白质组数据库(20207-2015.7.21)为参考进行检索,检索条件设置如下:蛋白水解酶设置为胰蛋白酶,最多允许两个漏切位点。甲硫氨酸氧化(M),蛋白质N-末端乙酰化,生物素标签(Biotin-N_3_-*O*-GlcNAc, *m/z*=994.3911)以及半胱氨酸巯基人为修饰物消除(-H_2_S, *m/z*=-33.9877)均设置为可变修饰。母离子的最大质量容差设置为2.0×10^-5^ mg/L,碎片离子的最大质量容差设置为0.5 Da,蛋白质和肽段谱图匹配的假阳性率(FDR)水平均设置为1%。

## 2 结果与讨论

### 2.1 半胱氨酸巯基-叠氮糖人为修饰物的构建

研究表明^[14]^,在碱性条件下,全乙酰化的非天然单糖可基于迈克尔加成原理与半胱氨酸的巯基发生反应,生成半胱氨酸巯基-叠氮糖人为修饰物。基于此,实验采用碳酸钠缓冲液(200 mmol/L, pH=10),利用Ac_4_GalNAz于37 ℃孵育巯基标准肽段90 min,构建半胱氨酸巯基-叠氮糖人为修饰物(见图1a)。孵育产物的MALDI-TOF MS分析结果如图1b所示,半胱氨酸巯基-叠氮糖人为修饰物(*m/z*=1464.694和*m/z*=1422.683)被成功检测。通常,Ac_4_GalNAz体外孵育巯基标准肽段的产物多为二乙酰化半胱氨酸巯基-叠氮糖人为修饰物(*m/z*=1464.694),在碱性条件下乙酰基发生部分水解,而产生单乙酰化的半胱氨酸巯基-叠氮糖人为修饰物(*m/z*=1422.683)。以上结果表明半胱氨酸巯基-叠氮糖人为修饰物的成功构建。

**图1:**
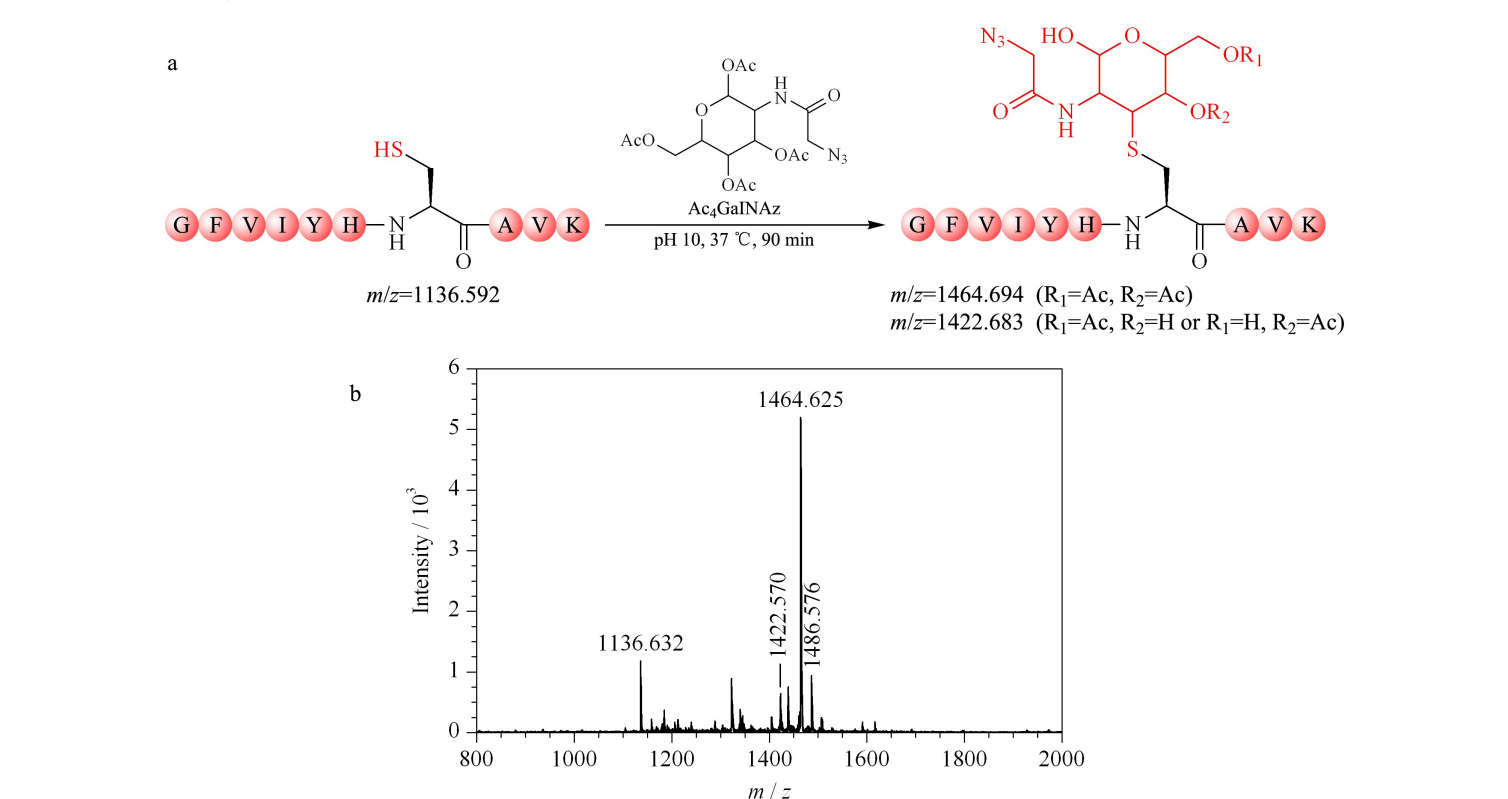
(a)Ac_4_GalNAz孵育巯基肽段的反应式和(b)反应产物的MALDI-TOF MS质谱图

### 2.2 半胱氨酸巯基-叠氮糖人为修饰物的氧化消除

MSH作为一种氧化及胺化试剂,可潜在用于半胱氨酸巯基修饰物的氧化消除。在此基础上,实验将上述构建的半胱氨酸巯基-叠氮糖人为修饰物作为底物,探究MSH氧化消除反应的最佳反应条件。如图2所示,在适宜的反应条件下,MSH可进攻半胱氨酸巯基-叠氮糖人为修饰物的亲核性硫原子,引发氧化消除反应,生成目标消除产物(*m/z*=1102.604)。为了避免强烈的碱性条件对*O*-GlcNAc糖基化修饰的破坏,实验首先确定了温和的磷酸钠缓冲液(50 mmol/L, pH=8)体系,并且MSH在该体系下具有较高的热稳定性^[21]^。由于升高反应温度有利于反应物分子吸收能量、促进反应的快速进行,实验考察了不同反应温度对MSH氧化消除半胱氨酸巯基-叠氮糖人为修饰物的影响。实验利用过量的MSH分别于4、20、50、80及95 ℃条件下处理半胱氨酸巯基-叠氮糖人为修饰物30 min(见表1)。MALDI-TOF MS分析结果(见附表1,详见http://www.chrom-China.com)显示,在4、20和50 ℃反应30 min后,半胱氨酸巯基-叠氮糖人为修饰物(*m/z*=1464.694和*m/z*=1422.683)依旧有较高的信号强度,且未发现产物峰,说明反应未发生;在80 ℃反应30 min后,目标消除产物峰(*m/z*=1102.604)部分生成,但半胱氨酸巯基-叠氮糖人为修饰物(*m/z*=1464.694和*m/z*=1422.683)仍存在,表明氧化消除反应不完全;而在95 ℃反应30 min后,半胱氨酸巯基-叠氮糖人为修饰物峰(*m/z*=1464.694和*m/z*=1422.683)完全消失,并且目标消除产物峰(*m/z*=1102.604)显示较高的信号强度,表明该半胱氨酸巯基-叠氮糖人为修饰物(*m/z*=1464.694和*m/z*=1422.683)已被完全氧化消除。上述实验结果表明,当温度达95 ℃时,MSH可较完全氧化消除半胱氨酸巯基-叠氮糖人为修饰物。实验进一步在95 ℃条件下评估了MSH氧化消除反应的反应时长(见表1)。MALDI-TOF MS分析结果显示,在95 ℃条件下缩短反应时间,目标消除产物(*m/z*=1102.604)部分生成,但半胱氨酸巯基-叠氮糖人为修饰物(*m/z*=1464.694和*m/z*=1422.683)仍存在,消除不完全(见附表2)。综上,实验采用95 ℃反应30 min的条件实现MSH快速高效地氧化消除半胱氨酸巯基-叠氮糖人为修饰物。

**图2:**
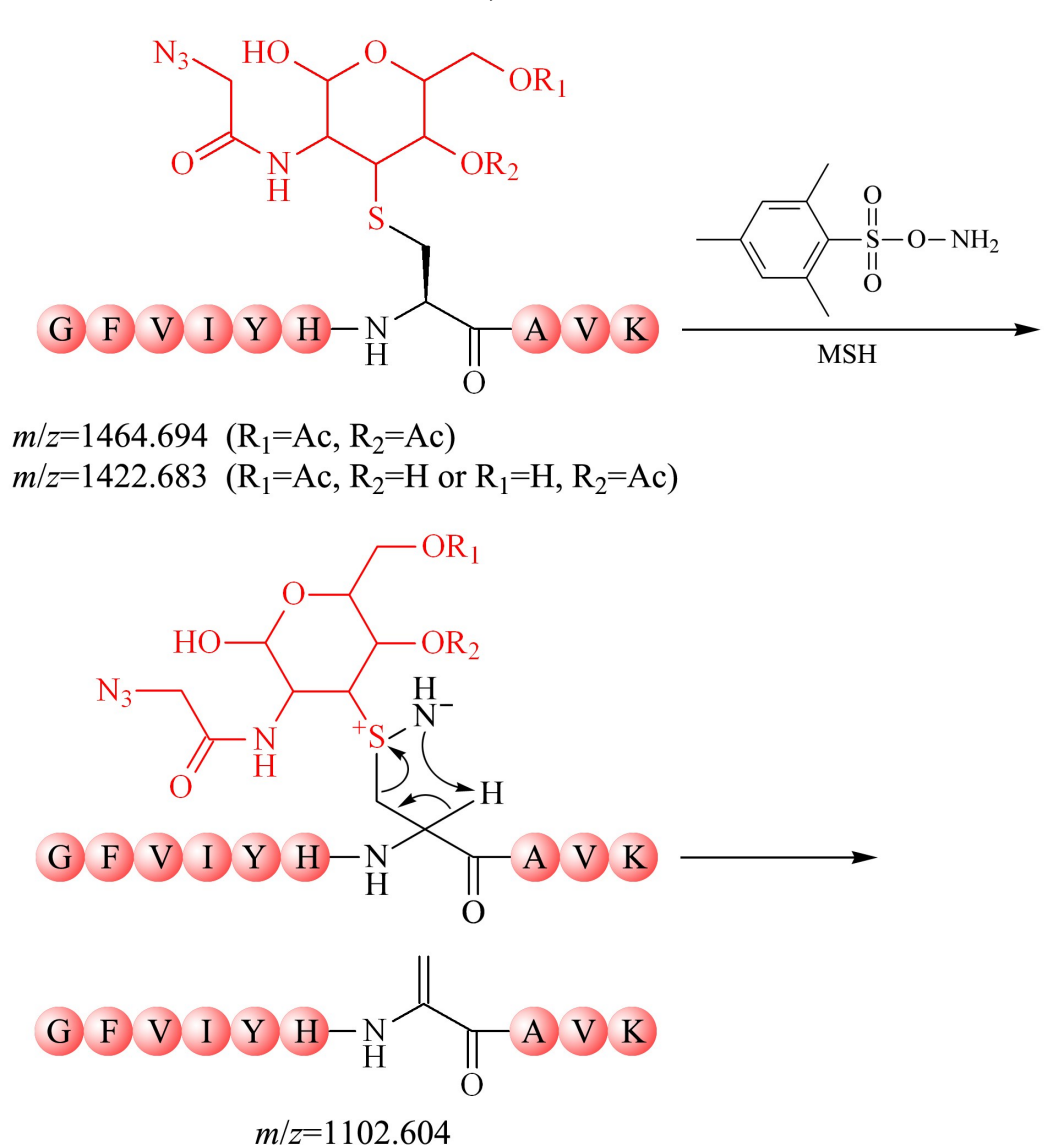
MSH氧化消除半胱氨酸巯基-叠氮糖人为修饰物机理

**表1:** MSH于不同条件下氧化消除半胱氨酸巯基-叠氮糖 人为修饰物的反应结果

pH	MSH/ (mmol/L)	Time/ min	Temperature/ ℃	Reaction
8.0	15	30	4	no
8.0	15	30	20	no
8.0	15	30	50	no
8.0	15	30	80	incomplete
8.0	15	30	95	complete
8.0	15	20	95	incomplete
8.0	15	10	95	incomplete

为了进一步验证MSH氧化消除法的可行性,实验制备了另一个半胱氨酸巯基-叠氮糖人为修饰物(*m/z*=1817.750) (见附图1),并利用MSH于95 ℃避光处理该半胱氨酸巯基-叠氮糖人为修饰物30 min,理论反应式如图3a所示;反应后的谱图如图3b所示,半胱氨酸巯基-叠氮糖人为修饰物(*m/z*=1817.750)完全消失,并生成目标消除产物(*m/z*=1455.661)。

**图3:**
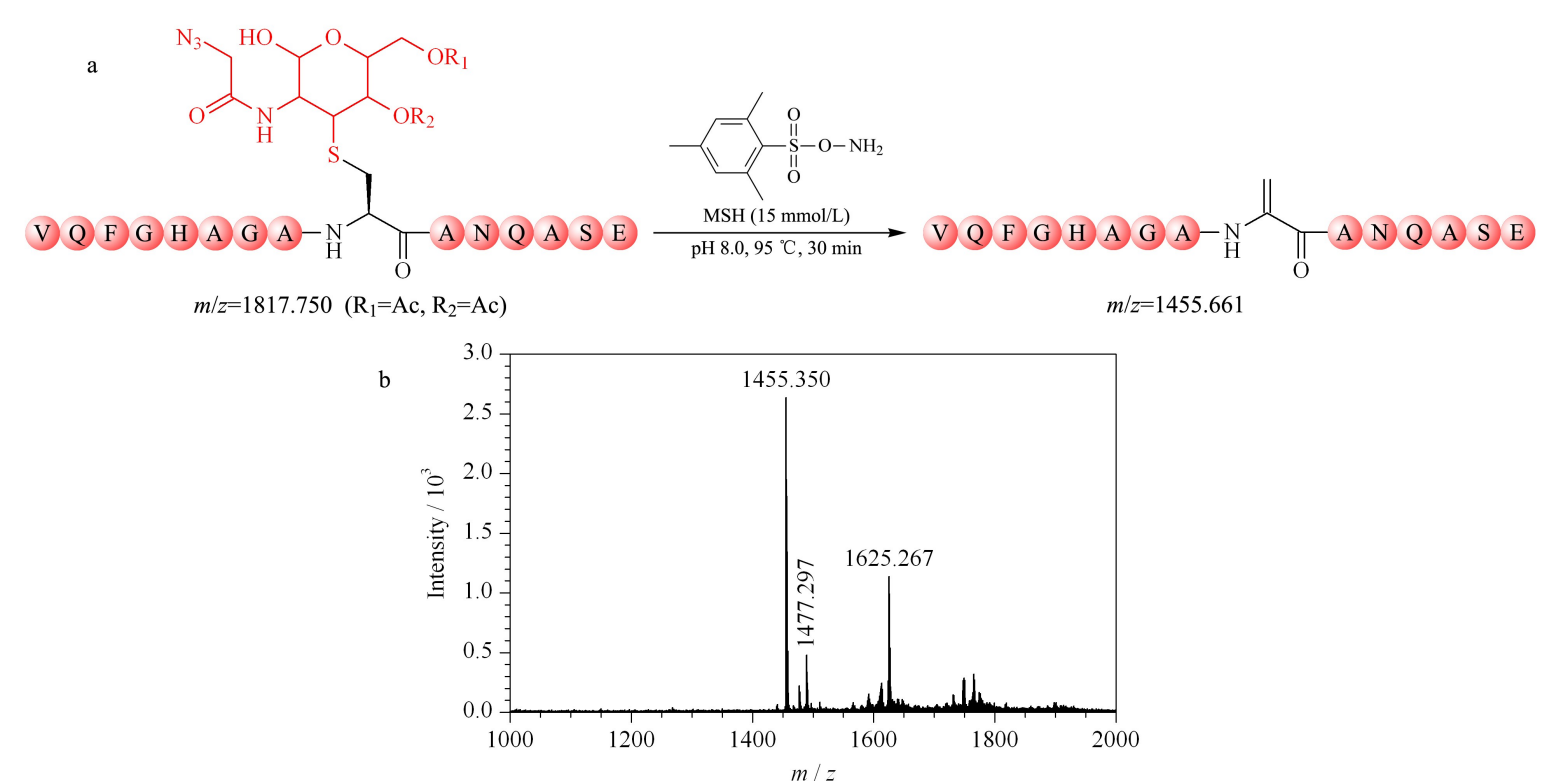
(a)MSH氧化消除半胱氨酸巯基-叠氮糖人为修饰物的反应式和(b)反应产物的MALDI-TOF MS质谱图

除此之外,图3b显示有未知峰(*m/z*=1625.267)的生成,该物质与推测的实验潜在副产物的相对分子质量均不匹配(见附图2)。由于反应体系复杂,未知峰(*m/z*=1625.267)的化学分子式难以确定,猜测其是由消除产物衍生形成的副产物。但是,由于未知物(*m/z*=1625.267)的相对分子质量与半胱氨酸巯基-叠氮糖人为修饰物及相关乙酰基水解峰的相对分子质量均不相同(见附图3),该未知物的存在并不会干扰*O*-GlcNAc糖基化修饰的质谱鉴定。综上,MSH氧化消除法简单可行,可高效去除半胱氨酸巯基-叠氮糖人为修饰物。

### 2.3 N_3_-*O*-GlcNAc修饰肽段的稳定性考察

为了考察MSH氧化消除反应是否会同时破坏*O*-GlcNAc糖基化修饰,实验选用了两条含N_3_-*O*-GlcNAc修饰的标准肽段(*m/z*=1333.758和*m/z*=1354.707) (见附图4)进行测试。两条N_3_-*O*-GlcNAc标准肽段分别经MSH在95 ℃条件下避光处理30 min,并采用MALDI-TOF MS分析。对比反应前、后的质谱图(见图4),两条N_3_-*O*-GlcNAc标准肽段峰经MSH处理后仍稳定存在,表明MSH氧化消除反应不会破坏N_3_-*O*-GlcNAc糖基化修饰。在后续复杂的生物代谢样本中,半胱氨酸巯基-叠氮糖人为修饰物与*O*-GlcNAc糖基化修饰肽段同时存在,该策略可在消除巯基-叠氮糖人为修饰物的同时,进一步保证内源*O*-GlcNAc糖基化修饰肽段在MSH氧化消除反应中的稳定性。因此,MSH氧化消除法可以应用于细胞中*O*-GlcNAc糖基化修饰肽段的富集鉴定。

**图4:**
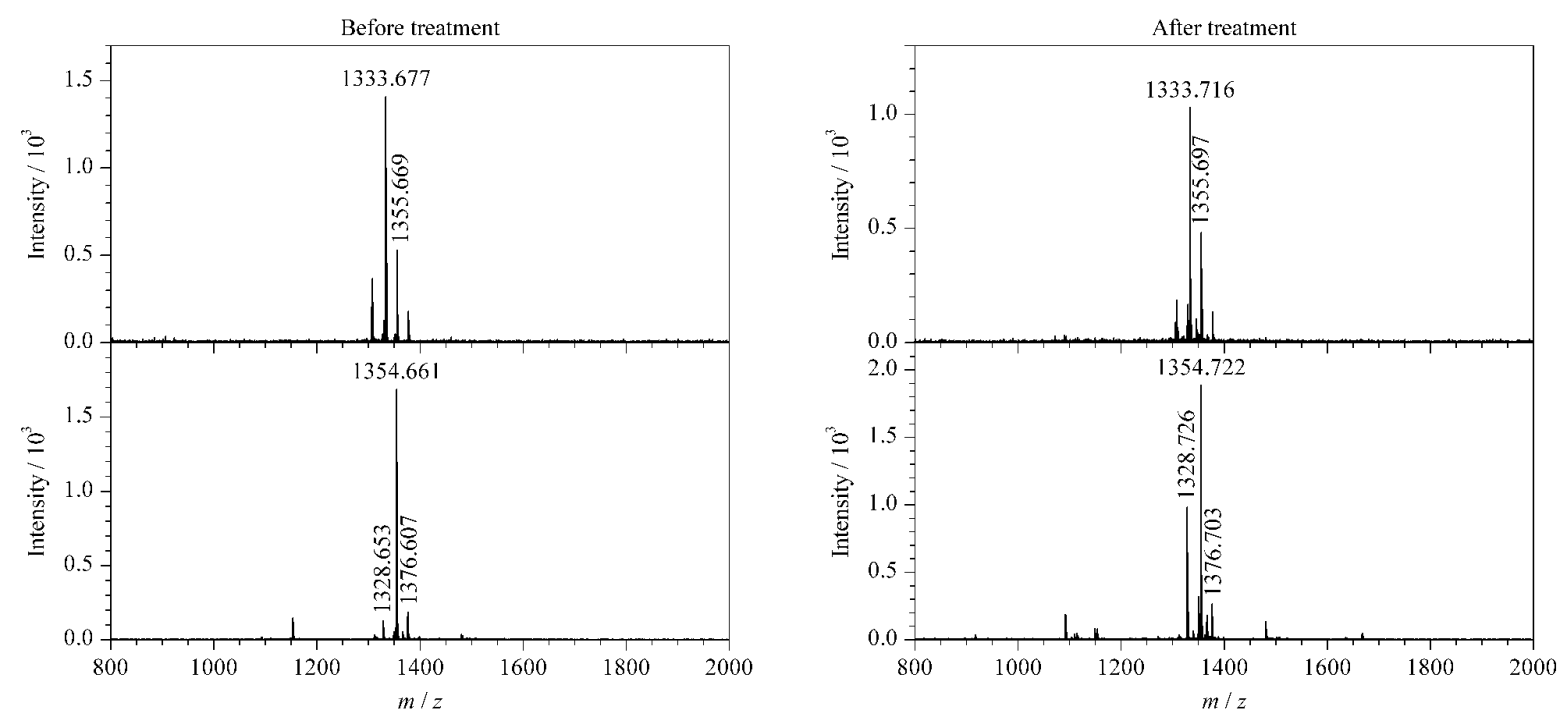
两条N_3_-*O*-GlcNAc肽段在MSH处理前、后的MALDI-TOF MS质谱图

### 2.4 *O*-GlcNAc修饰肽段的精准鉴定

*O*-GlcNAc糖基化修饰是一种动态可逆的翻译后修饰,它参与许多重要的生物信号通路。研究表明,细胞质和细胞核中的多种蛋白质会发生*O*-GlcNAc糖基化修饰^[22,23]^。为了深入了解*O*-GlcNAc糖基化修饰蛋白的生物学功能,实验选取Ac_4_GalNAz代谢Hela细胞后的核质蛋白作为研究对象。基于简单肽段层面,MSH特异性氧化消除半胱氨酸巯基-叠氮糖人为修饰物方法的成功构建,实验利用MSH氧化消除核质蛋白中的半胱氨酸巯基-叠氮糖人为修饰物,并鉴定到51条半胱氨酸巯基人为修饰消除产物肽段。该鉴定结果证明,在细胞代谢样本中,MSH氧化消除法可以去除半胱氨酸巯基-叠氮糖人为修饰物的假阳性干扰,从而实现*O*-GlcNAc糖基化肽段的精准鉴定。最终,实验利用生物素探针以及链霉亲和素磁珠富集鉴定核质蛋白中的N_3_-*O*-GlcNAc糖基化修饰肽段。通过质谱鉴定分析,在3次重复实验中成功富集鉴定到157条*O*-GlcNAc修饰肽段,归属于130个蛋白质。进一步的基因本体分析显示(见图5),富集的*O*-GlcNAc糖基化修饰蛋白主要分布于突触后密度、细胞质以及核染色体,主要参与细胞分裂、兴奋性突触后电位及微管运动等通路。其中,与转移酶活性、组蛋白乙酰转移酶活性以及微管结合等功能相关的*O*-GlcNAc糖基化修饰蛋白被显著富集,与文献^[8,24,25]^相符。以上结果表明,*O*-GlcNAc糖基化修饰蛋白广泛分布于细胞不同区室,在机体的多项生命活动中发挥着重要作用。

**图5:**
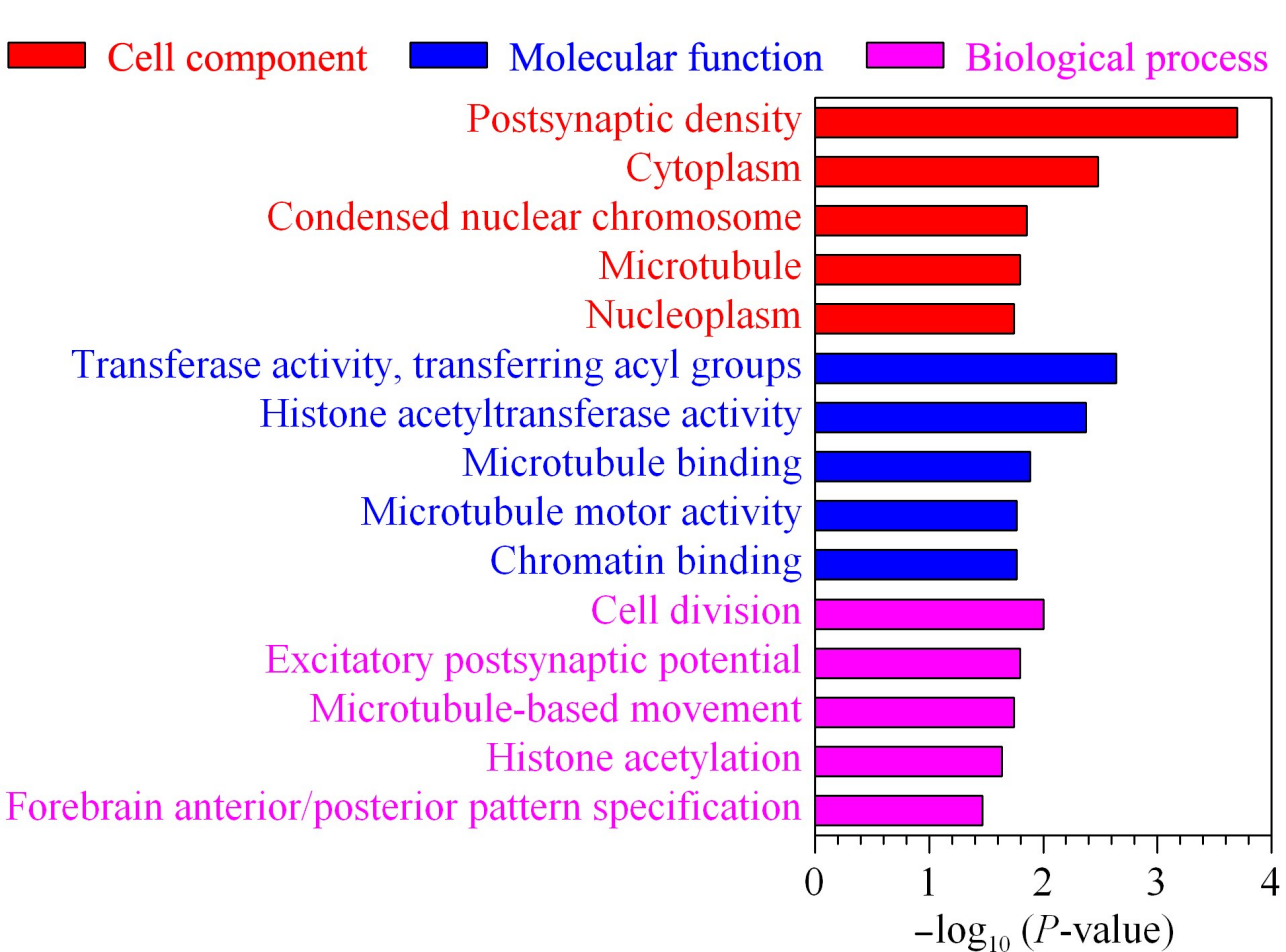
*O*-GlcNAc糖基化修饰蛋白的基因本体分析

## 3 结论

本研究发展了MSH特异性氧化消除半胱氨酸巯基-叠氮糖人为修饰物的方法,在保护*O*-GlcNAc糖基化修饰肽段的同时,特异性消除半胱氨酸巯基-叠氮糖人为修饰物。MSH氧化消除法操作简单,反应高效。该方法被成功应用于消除Ac_4_GalNAz代谢Hela细胞中产生的半胱氨酸巯基-叠氮糖人为修饰物,最终在Hela细胞核质蛋白中成功富集鉴定到157条*O*-GlcNAc修饰肽段,归属于130个蛋白质,并通过基因本体分析获取了*O*-GlcNAc糖基化修饰蛋白潜在的生物学功能。本研究成功消除了全乙酰化非天然单糖细胞代谢产物中的副产物,去除了其对*O*-GlcNAc糖基化修饰肽段富集鉴定的干扰,实现了细胞中*O*-GlcNAc糖基化修饰肽段的精准富集鉴定,为非天然糖代谢标记技术在糖蛋白组学分析中的应用提供了新的研究策略。

## References

[1] Hart G W. J Biol Chem, 2019, 294(7): 2211 30626734 10.1074/jbc.AW119.003226 PMC6378989

[2] Yang X, Qian K. Nat Rev Mol Cell Bio, 2017, 18(7): 452 28488703 10.1038/nrm.2017.22 PMC5667541

[3] Chatham J C, Zhang J, Wende A R. Physiol Rev, 2020, DOI: 10.1152/physrev.00043.2019 PMC8428922 32730113

[4] Yang Y R, Jang H J, Choi S S, et al. Diabetologia, 2015, 58(12): 2867 26342595 10.1007/s00125-015-3736-z

[5] Lefebvre T, Guinez C, Dehennaut V, et al. Expert Rev Proteomic, 2005, 2(2): 265 10.1586/14789450.2.2.265 15892570

[6] de Queiroz R M, Carvalho E, Dias W B. Front Oncol, 2014, 4:132 24918087 10.3389/fonc.2014.00132 PMC4042083

[7] Li J, Tan Z P. Chinese Journal of Biochemistry and Molecular Biology, 2020, 36(9): 987

[8] Zachara N, Akimoto Y, Hart G W. Essentials of Glycobiology. 3rd ed. New York: Cold Spring Harbor Laboratory Press, 2017

[9] Laughlin S T, Bertozzi C R. Proc Natl Acad Sci U S A, 2009, 106(1): 12 19104067 10.1073/pnas.0811481106 PMC2629201

[10] Zachara N E, Hart G W. Biochim Biophys Acta-Mol Cell Biol Lipids, 2006, 1761(5/6): 599 10.1016/j.bbalip.2006.04.007 16781888

[11] Darabedian N, Pratt M R. Methods in Enzymology. New York: Academic Press, 2019, 622:293 31155058 10.1016/bs.mie.2019.02.017 PMC6860911

[12] Chuh K N, Batt A R, Pratt M R. Cell Chem Biol, 2016, 23(1): 86 26933738 10.1016/j.chembiol.2015.11.006 PMC4779183

[13] Shi J. Chinese Journal of Biochemistry and Molecular Biology, 2020, 36(6): 609

[14] Qin K, Zhang H, Zhao Z, et al. J Am Chem Soc, 2020, 142(20): 9382 32339456 10.1021/jacs.0c02110

[15] Qin W, Qin K, Fan X, et al. Angew Chem, 2018, 130(7): 1835

[16] Hao Y, Fan X, Shi Y, et al. Nat Commun, 2019, 10(1): 1 31492838 10.1038/s41467-019-11942-y PMC6731260

[17] Qin W, Qin K, Zhang Y, et al. Nat Chem Biol, 2019, 15(10): 983 31332308 10.1038/s41589-019-0323-5

[18] Matsuo J, Kozai T, Ishibashi H. Org Lett, 2006, 8(26): 6095 17165938 10.1021/ol062620w

[19] Bernardes G J L, Chalker J M, Errey J C, et al. J Am Chem Soc, 2008, 130(15): 5052 18357986 10.1021/ja800800p

[20] Zemskov I, Kropp H M, Wittmann V. Chem-Eur J, 2016, 22(31): 10990 27346324 10.1002/chem.201601660

[21] Mendiola J, Rincón J A, Mateos C, et al. Org Process Res Dev, 2009, 13(2): 263

[22] Tian J, Ma X Y, Wang Z P. Chemistry of Life, 2018, 38(5): 673

[23] Comer F I, Hart G W. J Biol Chem, 2000, 275(38): 29179 10924527 10.1074/jbc.R000010200

[24] Zhao L, Li M, Wei T, et al. Int J Mol Sci, 2020, 21(1): 173

[25] Love D C, Hanover J A. Sci Signaling, 2005, 2005(312): 13 10.1126/stke.3122005re13 16317114

